# How copy number variations shape brain developmental disorders: Unraveling the synaptic puzzle

**DOI:** 10.1111/pcn.70009

**Published:** 2025-12-23

**Authors:** Tianqi Wang, Kubra Trabzonlu, Emily Fullard Jones, Yasir Ahmed Syed

**Affiliations:** ^1^ Neuroscience and Mental Health Innovation Institute, Hadyn Ellis Building Cardiff University Cardiff UK; ^2^ School of Bioscience, Cardiff University Cardiff UK

**Keywords:** copy number variation (CNVs), diseases models, drug discovery, neuronal development disorders (NDDs), synapse development

## Abstract

Neurodevelopmental disorders (NDDs), such as schizophrenia (SCZ), Attention‐deficit/hyperactivity disorder (ADHD), autism spectrum disorder (ASD), learning disabilities, and intellectual disabilities (ID), are highly prevalent. One significant genetic factor associated with NDDs is copy number variations (CNVs), which are structural changes in the genome that involve deletions or duplications of DNA segments. CNVs are known to significantly elevate the risk of developing NDDs and are increasingly being studied for their role in these conditions. While CNVs encompass a wide range of genetic alterations, emerging evidence suggests they may disrupt key biological processes, such as synaptic development and function in the brain, which are critical for learning and behavior. This review synthesizes findings from genetics, molecular biology, and related fields to explore the link between CNVs and synaptic pathology with therapeutic investigations. By understanding how CNVs compromise synaptic function, we identify paths to more targeted and effective therapies for neurodevelopmental disorders associated with CNVs.

Neurodevelopmental disorders (NDDs) affect the development of the brain and nervous system and affect around 15% of children and adolescents worldwide.^1^ Synaptic development and function dysfunction contributes to neurodevelopmental and neuropsychiatric disorders including ASD, SCZ, and ID.^2^ Copy number variations (CNVs) are a significant type of genetic variation linked to neurodevelopmental disorders. These structural alterations, which involve the deletion or duplication of genomic regions, can affect brain development by altering gene dosage, disrupting critical pathways, and impairing neuronal connectivity. Genes involved in synaptic development and function are particularly interesting when affected by CNVs, given their crucial role in establishing and maintaining neuronal networks. Therefore, CNVs offer valuable models for understanding how genetic variations contribute to neurodevelopmental disorders through synaptic dysfunction. This review examines the complex relationship between CNVs and synaptic development, clarifying the shared and distinct mechanisms by which these CNVs disrupt synaptic processes. It emphasizes the significant impact of CNVs on synaptic networks and their relevance to human health and disease (Fig. 1).

**Fig. 1:**
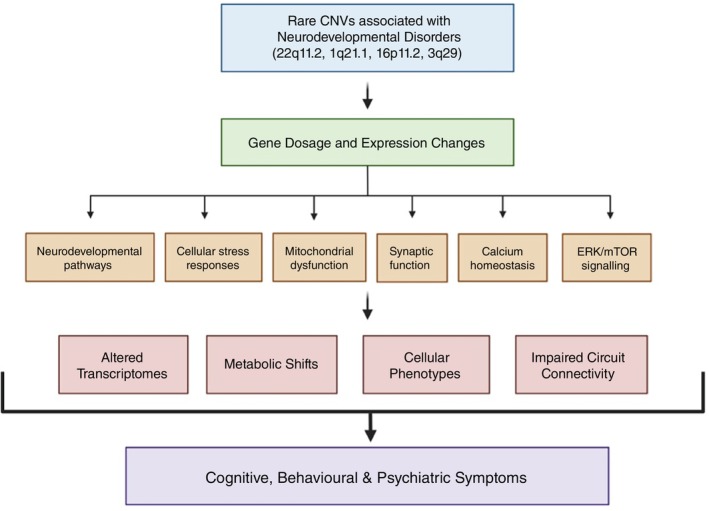
This schematic diagram illustrates the mechanistic cascade through which rare copy number variations (CNVs) associated with neurodevelopmental disorders (22q11.2, 1q21.1, 16p11.2, and 3q29) may drive cognitive, behavioral, and psychiatric symptoms. The top section highlights these focal CNVs, which are strongly linked to conditions such as autism spectrum disorder (ASD) and schizophrenia. CNVs directly impact the dosage and expression of critical genes, marking the initial molecular disruption that triggers widespread dysregulation. Key pathways affected include Neurodevelopmental pathways: Governing neuronal differentiation, migration, and circuit formation. Cellular stress responses: Coping mechanisms for oxidative, metabolic, or proteotoxic stress. Mitochondrial dysfunction: Impairments in energy production and calcium buffering in neurons. Synaptic function: Defects in transmission, plasticity, and structural integrity (e.g., dendritic spines, synaptic vesicles). Calcium homeostasis: Dysregulated intracellular calcium signaling, essential for plasticity and excitability. ERK/mTOR signaling: Pathways regulating cell growth, protein synthesis, and synaptic plasticity. Dysregulation of these pathways leads to four core alterations: Altered Transcriptomes: Widespread changes in gene expression across neurons and glia. Metabolic Shifts: Rewiring of energy metabolism due to mitochondrial or stress pathway defects. Cellular Phenotypes: Morphological and functional abnormalities in neurons (e.g., dendritic arborization issues, synaptic loss) and glia. Impaired Circuit Connectivity: Disruption in synaptic wiring and communication within cortical, limbic, and thalamic circuits. These combined disruptions ultimately result in clinical features such as intellectual disability, social deficits, repetitive behaviors, and psychosis. This diagram provides a comprehensive overview of how CNVs disrupt brain development and function, highlighting both shared mechanisms (e.g., synaptic and calcium pathway involvement) and CNV‐specific contributions to the pathology of neurodevelopmental disorders.

## The Synapse System

### Synapse structure and function

Healthy brain function depends on proper synaptic organization and reliable neuronal communication throughout all stages of brain development. Synaptic transmission happens in two main ways: electrical synapses, which allow for direct ion flow, and chemical synapses, which involve the release of neurotransmitters.^3^ Furthermore, synapses are categorized as either excitatory, promoting neuronal firing, or inhibitory, which suppress it. The principal neurotransmitters mediating these activities are glutamate at excitatory synapses and γ‐aminobutyric acid (GABA) at inhibitory synapses. Excitatory synapses are mainly found on dendritic spines, whereas inhibitory synapses are generally located on cell bodies and proximal axons.^4^ A typical synapse structure consists of a presynaptic terminal, carrying neurotransmitter vesicles, and the postsynaptic structure, which receives the neurotransmitters *via* surface receptors. These two domains are separated by a narrow gap‐ the synaptic cleft. The formation of a functional synapse is a highly coordinated, multi‐step process. The initial contact between pre‐ and postsynaptic neurons is mediated by cell adhesion molecules, such as neurexins (presynaptic) and neuroligins (postsynaptic).^5^ The C‐terminus of neuroligins plays a substantial role in postsynaptic activity because it facilitates the binding of AMPA and NMDA receptors, key components of excitatory synaptic signaling.^6^ Following initial adhesion, motor proteins, dynamins, and kinases transport synaptic components along microtubule networks in axons and dendrites. This trafficking allows for the assembly and modulation of functional synapses. Scaffolding proteins and kinase‐mediated signaling further support synapse maturation.^2^

During early neuronal development, the presynaptic structure forms and quickly matures, becoming functional and organized. Despite variations in synaptic phenotypes, presynaptic structures remain similar across organisms. Presynaptic terminals, the active regions, contain synaptic vesicles (SVs) that fuse with the presynaptic membrane in response to Ca2+ influx through voltage‐gated calcium channels, releasing neurotransmitters into the synaptic cleft.^7^ The presynaptic compartment hosts, including GTPases, calcium channels, ionic transporters, axonal trafficking proteins and SNARE complexes.^8^

#### Presynaptic machinery

The primary function of the presynaptic terminal is to facilitate the release of neurotransmitters in response to neuronal activity.^7^ This process is mediated by synaptic vesicles (SVs), which constitute the core machinery of presynaptic function.^7^ Presynaptic function begins with the activation of voltage‐gated calcium channels (VGCCs), triggered by fluctuations in intra‐ and extracellular calcium levels, leading to an influx of Ca^2+^ into the cell.^8^ This Ca^2+^ influx triggers SVs to be loaded with neurotransmitters *via* the activity of v‐type ATPase (v‐ATPase), which acidifies the lumen of the SVs to support neurotransmitter uptake.^9^ v‐ATPase acidifies SV lumens to support neurotransmitter uptake.^10^

Synaptic vesicles (SVs) bind to presynaptic active zone proteins to dock for endocytosis. Their pool is replenished *via* context‐dependent pathways,^9^ and retrieved SVs are stored in reserve or recycling pools. Reserve pool vesicles are mobilized by action potentials, undergoing docking, priming, and fusion to release neurotransmitters *via* exocytosis. This fusion process is mediated by the SNARE complex, which includes VAMP2, syntaxin‐1, and SNAP25.^11^

#### Assembly and maturation of the postsynaptic density

Excitatory glutamatergic synapses are typically localized on dendritic spines, which are protrusions extending from dendrites.^12^ These spines house the postsynaptic density (PSD), a dense and highly organized protein complex situated beneath the postsynaptic membrane.^13^ The PSD complex regulates the localization, clustering and function of synaptic receptors and signaling molecules, comprising cytoskeletal proteins, scaffold proteins, ionotropic receptors, signaling enzymes and trafficking proteins.^14^ The upper (exterior) layer of the PSD is enriched with ionotropic glutamate neurotransmitter receptors, such as stable NMDA receptors and soluble AMPA receptors, along with membrane adhesion proteins including N‐cadherin, neuroligins (NLGNs), leucine‐rich repeat transmembrane proteins (LRRTMs) and Eph receptors. These proteins mediate Ca^2+^ influx and maintain synaptic alignment by bridging pre‐ and post‐synaptic compartments.^15^ Membrane‐associated guanylate kinase (MAGUK), guanylate kinase‐associated protein (GKAP) SH3, multiple ankyrin repeat domains protein (SHANK) and HOMERs are core scaffolding proteins located beneath neurotransmitter receptors and adhesion molecules within the PSD, where they facilitate extensive protein–protein interactions. The MAGUK protein superfamily consists of DLG1, DLG2, DLG3 and DLG4 (PSD‐95) sub‐families, all of which contain PSD‐95/Discs large/Zona occludens‐1 (PDZ) domains that mediate binding to either NMDA receptors or NLGNs.^16^, ^17^ Among these, PSD‐95 is a particularly important scaffold protein within the PSD complex, contributing critically to synaptic plasticity. The GKAP family interacts with PSD‐95 through its guanylate kinase (GK) domain, forming a bridge to SHANK proteins, which serve as central scaffolding elements of the PSD.^18^ The SHANK family, often described as master organizer of the PSD, interacts with HOMER proteins and actin cytoskeleton proteins, thereby linking the PSD to metabolic glutamate receptors (mGluRs).^19^

Several kinases and GTPase signaling molecules are embedded within the PSD. One key example is calcium/calmodulin‐dependent protein kinase II (CaMKII), the most abundant kinase in the PSD, essential for long‐term potentiation (LTP) and its interaction with the GluN2B subunit of NMDA receptors.^20^ The phosphorylation of synaptic GTPase‐activating protein (SYNGAP) initiates the activation of Ras GTPase complexes, and SYNGAP directly interacts with PSD‐95.^15^ Another GTPase, Kalirin‐7, also associated with PSD‐95 and its activation promotes F‐actin polymerization, which influences dendritic spine morphology and synaptic stability.^20^ The PSD core also includes activity regulated cytoskeleton‐associated protein (ARC), CaMKIIß and ß‐catenin.^15^ The ARC complex aids in NMDA receptor endocytosis, while ß‐catenin is crucial for synaptic development. The core PSD structure also includes the fragile X mental retardation protein (FMRP) and its partner, CYFIP1, which regulate the translation of other PSD‐associated proteins.^21^, ^22^

The postsynaptic density (PSD) exhibits structural, molecular, and biochemical diversity across brain regions like the cerebral cortex, midbrain, and cerebellum. In the hippocampus, the PSD is rich in phosphorylated peptides, kinases, and phosphatases, highlighting its developmental plasticity.^15^ During embryonic development in mice, PSD maturation follows a sequential process: GKAP binds to PSD‐95 first, followed by SHANK protein incorporation, reflecting dynamic synaptogenesis.^23^ Wnt signaling plays a key role in synapse assembly, supporting intracellular communication. Broader neurotransmitter pathways also influence early and mature cortical development. Genes like *DTNBP1* and its effector *AGAP1* regulate dendritic spine morphology and endosomal trafficking, essential for normal synaptic function.^24^

### CNVs linked to synaptic development

**Fig. 2:**
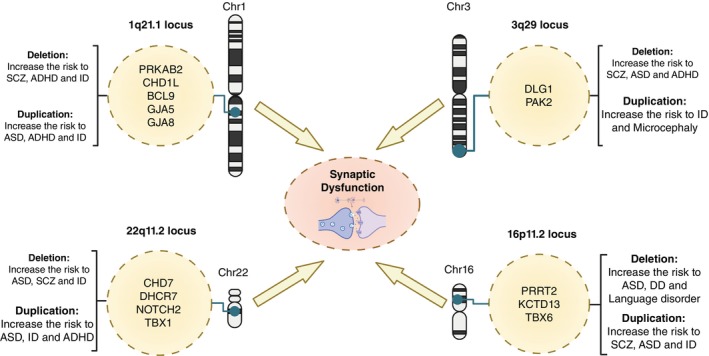
Impact of Copy Number Variation on Synaptic Dysfunction. Deletion or duplication of specific chromosomal regions ‐ 1q21.1 (Chr1), 22q11.2 (Chr22), 16p11.2 (Chr16), and 3q29 (Chr3) ‐ is significantly linked to an increased risk for developmental and psychiatric disorders. Resources like GeneReviews® indicate that these genomic areas contain critical risk genes contributing to abnormal brain development, potentially converging on mechanisms that lead to synaptic dysfunction. ADHD, Attention Deficit Hyperactivity Disorder; ASD, Autism Spectrum Disorder; DD, Developmental Disability; ID, Intellectual Disability; SCZ, Schizophrenia.

Disruptions in synaptic processes are increasingly recognized as contributing factors in the pathogenesis of psychiatric and neurodevelopmental disorders. Copy number variations (CNVs), by altering gene dosage, provide a direct link between genetic variation and synaptic dysfunction (Fig. 2). CNVs have been strongly associated with neurodevelopmental disorders, including ASD, ID, and SCZ.^25^, ^26^, ^27^ Although CNVs linking to psychiatric disorder phenotypes are quite rare in the population, their odds ratios for high‐risk factors in ASD and SCZ range from 2 to 60.^28^ Deletion CNVs associated with SCZ are highly enriched with synaptic network proteins. These include synaptic adhesion and scaffolding proteins, as well as NMDA receptors and ARC protein complexes.^29^, ^30^, ^31^, ^32^

Studies using animal models and cortical organoids have revealed that CNVs also affect key neurodevelopmental processes, including neuronal proliferation and neuronal migration.^27^, ^33^ Moreover, multiple CNVs converge on common molecular pathways, particularly those related to synaptic function and plasticity.^34^, ^35^ This convergence may account for the overlapping phenotypes observed across different neurodevelopmental disorders.^36^, ^37^ Network and gene‐set analyses confirm *de novo* CNVs enrich for synaptic signaling, chromatin regulation, and MAPK pathways.^38^ ASD‐linked deletion CNVs impair synaptic function, while both deletions and duplications target postsynaptic glutamatergic synapse genes.^30^, ^38^ with large‐scale studies validating synaptic and neurobehavioral gene enrichment.^28^

Gene set enrichment analysis of CNVs underlying the pathogenesis of SCZ and ASD showed common pathways including synaptic function, fragile X mental retardation protein targets and chromatin regulation.^30^, ^38^, ^39^, ^40^ Another study revealed that four gene sets related to synaptic pathways were enriched in individuals with copy number variations (CNVs) linked to schizophrenia (SCZ) and autism spectrum disorder (ASD). Furthermore, study involving parent–child trios found a similar enrichment of synaptic proteins in people with SCZ and *de novo* CNVs.^30^ Integrating neuroimaging, cognitive, and molecular data is crucial for understanding how CNVs lead to neurodevelopmental disorders and identifying potential therapeutic targets.^33^, ^35^

### CNVs‐related disease models

Copy number variation (CNV), a genomic imbalance, is strongly linked to the etiology of neuropsychiatric disorders like schizophrenia and autism.^33^ To study CNVs and their effects on synaptic development, various models have been created. These include CNV‐related mouse models, 2D neuronal models, and 3D brain organoids, each offering distinct yet overlapping insights into synaptic development in these disorders.^41^, ^42^ CNVs cause pathogenic effects through mechanisms like gene dosage effects, positional effects, and disruption of topological associated domain (TAD) boundaries. This ultimately disrupts the expression of neurodevelopmental genes crucial for synaptic formation, such as *SHANK3* and *NRXN1*.^38^ Interestingly, maternal CNV variants can also pass epigenetic marks (e.g., DNA methylation) to offspring, leading to autism‐like behaviors even without direct CNV inheritance.^38^

Mouse models demonstrate *in vivo* phenotypes such as region‐specific changes in synaptic density and excitation/inhibition imbalances.^43^, ^44^, ^45^ Meanwhile, 2D neuronal systems, derived from induced pluripotent stem cells (iPSCs), provide critical insights into synapse number, dendritic architecture, and synaptic protein expression. These models help study syndromes and loss‐of‐function mutations.^42^, ^46^, ^47^, ^48^ In parallel, 3D organoid models have advanced our understanding of human‐specific neurodevelopmental processes like cortical folding, neuronal migration, and excitation/inhibition balance. Across these systems, common synaptic phenotypes emerge, reinforcing that each model offers unique yet complementary insights into the cellular and circuit‐level mechanisms underlying neuropsychiatric disorders. Using multiple model systems facilitates cross‐validation and improves the translational applicability of findings. For example, both 2D neuronal models^46^ and CNV‐related mouse models^49^ show changes in synaptic density and dendritic spine morphology across genetic backgrounds. Similarly, excitatory/inhibitory imbalances have been reported in CNV‐related mouse models,^43^ 2D neuronal models,^50^ and 3D organoid models.^51^ All models have also shown calcium signaling abnormalities.^52^, ^53^

However, each model also has species‐specific differences in developmental timing, gene expression patterns, structural complexity, and cellular diversity. Human neuronal models (both 2D and 3D) often develop slower than mouse models.^27^, ^41^, ^42^, ^46^, ^47^, ^50^, ^52^, ^54^, ^55^, ^56^, ^57^ Additionally, some risk genes for neuropsychiatric disorders display species‐specific expression patterns or functions.^48^ 3D organoid models are particularly valuable because they can recapitulate structural features of the human brain not fully represented in mouse models or 2D cultures.^27^, ^51^, ^54^ These models also enable the exploration of cellular heterogeneity, capturing a broader range of neuronal subtypes and states, including shifts in cell‐type proportions in CNV‐associated conditions compared to mouse models or 2D cultures. The integrity of synaptic structure and function is fundamental for neural signal transmission. CNVs is likely to contribute to neurodevelopmental disorders by disrupting these crucial processes.

## Genes and CNVs Involved in Synaptic Development

### Synapse formation and maturation

CNVs can compromise the establishment and development of synapses by perturbing key molecular processes such as protein expression, neuronal adhesion, and axon guidance. Disruptions in these fundamental processes can adversely affect synaptic structure and function, thereby impeding neural circuit formation.

The 16p11.2 deletion/duplication affects synapse formation and dendritic spine morphology by altering the expression of synaptic proteins such as MAPK3 and MVP.^58^ *MAPK3* encodes the mitogen‐activated kinase *ERK1*, a pivotal regulator of synaptic development that modulates dendritic spine density *via* the *ERK1/2* signaling pathway.^59^, ^60^ In rodent models, *ERK1/2* activation is required for brain‐derived neurotrophic factor (BDNF)‐induced synaptic remodeling; the loss of *MAPK3* impairs BDNF's ability to promote dendritic spine formation, thereby highlighting the role of this pathway in synaptic maturation.^59^ *TAOK2*, a serine/threonine kinase within the 16p11.2 locus, is also crucial for the stability and maturation of dendritic spines. This phenotype is validated in hippocampal CA1 region of 16p11.2 deletion mice,^43^ iPSC cortical neurons^58^ and 60‐day differentiated cortex organoids.^27^ Notably, organoids also exhibit synergistic effects of abnormal cortical lamination and reduced synaptic connectivity.^27^ It phosphorylates of *Septin7* (*Sept7*), promoting its translocation to dendritic spines, where it interacts with the scaffolding protein *PSD95* to facilitate proper glutamate receptor localization and synaptic assembly.^61^ Loss of *TAOK2* impairs *Sept7* phosphorylation, leading to unstable dendritic protrusions, ectopic shaft synapses, and disrupted NMDA receptor‐mediated Ca^2+^ compartmentalization, all of which are essential for proper synaptic signaling.^61^

The 22q11.2 deletion impacts synaptic vesicle cycling and neurotransmitter release by disrupting the function of *SEPT5* and *SNAP29*.^62^ *ZDHHC8*, another gene in this region, encodes a palmitoyl acyltransferase (PAT) enzyme involved in protein palmitoylation, a post‐translational modification essential for synaptic protein trafficking and membrane localisation.^63^, ^64^ In mouse models, *ZDHHC8* localizes to vesicle‐like structures along dendrites and is implicated in dendritic spine maturation and neurotransmission.^63^ The mice exhibit altered protein distribution, diminished responses to the NMDA receptor antagonist *MK‐*801, and impaired synaptic transmission.^63^ Earlier studies further demonstrate that *ZDHHC8* loss reduces palmitoylation of proteins required for axonal growth and synaptic integrity; these deficits are rescued by reintroducing function *ZDHHC8*.^63^

The 1q21.1 deletion/duplication affects synaptic density and neuronal maturation *via* altered calcium signaling pathways.^41^ The human‐specific SRGAP2C gene, a variant of the *SRGAP2* gene family, located near this locus, plays a key role in the development of dendritic spines. While the original *SRGAP2* gene helps spines mature, *SRGAP2C* slows down this process. This leads to a higher number of immature spines with long necks, a characteristic of human synaptic neoteny.^65^

The 3q29 CNV locus contains several genes implicated in synaptic development, including *DLG1*, a *PSD‐95* homolog.^66^ *DLG1* is integral to postsynaptic architecture, interacting with glutamate receptors, adhesion molecules, and cytoskeletal components to maintain synaptic function.^66^ In Drosophila, the novel *dlg1* tool allows visualization of *DLG1* at synapses without perturbing baseline physiology, confirming its role in activity‐dependent plasticity across cholinergic and glutamatergic synapses.^66^ Loss of function mutations in *DLG1* lead to enlarged glutamate receptor fields, increased synaptic bouton size, and disrupted postsynaptic density organization.^66^ Electrophysiological data show that these structural changes reduce neurotransmitter release probability by mis localizing voltage‐gated Ca^2+^ channels, resulting in deficits in short term plasticity.^66^ Baba *et al*. (2019)^67^ observed increased cortical excitability in a 3q29 deletion mouse model. In these mice, the firing frequency of excitatory neurons increased 2.3‐fold. Additionally, the functional defects in AMPA receptors observed in human iPSC neurons provide further insight into the molecular mechanism behind this phenotype. These findings suggest that DLG1 could be one of key genes through which 3q29 CNV impacts synaptic function. *PAK2* haploinsufficiency in mice reduces spine density and impairs long‐term potentiation (LTP), primarily *via* disrupted LIMK1‐cofilin signaling.^68^ This is primarily attributed to disruptions in the LIMK1‐cofilin signaling pathway, which is critical for actin polymerization and synaptic structure maintenance.^68^ Notably, Drosophila models indicate that combined haploinsufficiency of *DLG1* and *PAK2* is necessary to elicit synaptic deficits, suggesting synergistic effects.^69^

Several additional CNVs further illustrate the diversity of mechanisms influencing synapse formation. Deletion of *NRXN1* disrupts trans‐synaptic signaling, impairing presynaptic organization,^70^ while *SHANK2* deletion alters postsynaptic density organization and glutamatergic signaling.^71^ *CNTN4* CNVs interfere with neuronal cell adhesion, affecting axon guidance and synapse formation.^70^ The *DLG1* and *DLG2* CNVs impact postsynaptic scaffolding through altered synaptic protein clustering^72^ and *ASTN2* CNVs affect neuronal migration and synapse formation by potentially disrupting synaptic adhesion.^73^ Finally, *UBE3A* duplications affect synaptic protein turnover by altering ubiquitin‐mediated protein degradation pathways.^70^ Collectively, these observations underscore the importance of precise synaptic formation and maturation for maintaining neural circuit integrity. Table 1 presents four rare pathogenic CNVs, their associated neuronal and synaptic genes, related synaptic phenotypes, and neurodevelopmental disorder.

**Table 1:** Rare pathogenic CNVs: neuronal/synaptic genes, neurodevelopmental disorders, and synaptic phenotypes.

CNVs	Effected genes	Synaptic phenotypes	Associated disorders
3q29 deletion/duplication syndrome	*PAK2, DLG1, FBXO45, NCBP2*	Enhancement on excitatory neuronal activity in cerebral cortex	SCZ, ASD, ADHD, Intellectual disability, Anxiety, Macrocephaly/microcephaly
16p11.2 deletion/duplication syndrome	*TAOK2, KCTD13, MAPK3, SH2B1, DOC2A, TBX6, SEZ6L2, PRRT2*	Impairment on calcium homeostasis Reduction on neuronal firing rate, synchronicity, and oscillation Less excitatory synapses with enhanced synaptic strength Hyperexcitability on GABAergic synaptic transmission	Delayed development, Epilepsy, ADHD, ASD, SCZ, Depression
22q11.2 deletion/duplication syndrome	*SEPT5, MAPK1, DGRC8, ZDHHC8, PRODH*	Abnormal axonal outgrowth Abnormal calcium homeostasis Alterations on inhibitory synaptic events Dysregulation on inhibitory and excitatory neuronal networks	Delayed development, Intellectual disability, ADHD, ASD
1q21.1 deletion/duplication syndrome	*BCL9, CHD1L, HYDIN1L*	Aberrations on neuronal network and calcium activity Impairment on synaptogenesis Enhancement on dopamine cell firing and neuronal activity	ASD, ADHD, SCZ, Delayed development

ADHD, Attention Deficit Hyperactivity Disorder; ASD, Autism Spectrum Disorder; SCZ, Schizophrenia.

### Synaptic Pruning and Connectivity

CNVs interfere with the refinement of neural circuits by disrupting synaptic pruning and connectivity, processes essential for optimizing brain function. Aberrations in these mechanisms can lead to maladaptive connectivity patterns that are frequently implicated in neuropsychiatric disorders.

The 16p11.2 deletion/duplication alters synaptic density and neuronal connectivity disrupting RhoA signaling and the actin cytoskeleton.^58^ A key gene within this locus, *KCTD13*, encodes a protein that modules synaptic transmission through its inhibitory regulation of RhoA, a small GTPase essential for cytoskeletal organization and synaptic plasticity.^74^, ^75^ Knockout studies in mice demonstrate that *KCTD13* loss results in elevated RhoA activity, leading to pronounced structural and functional deficits, including reduced dendritic length and complexity, decreased spine density, and a lower number of functional synapses.^74^ Electrophysiological analyses reveal a significant reduction in the frequency of miniature excitatory postsynaptic currents (mEPSCs), with unchanged amplitude, indicating a presynaptic deficit rather than alterations in postsynaptic receptor function.^74^ Importantly, pharmacological inhibition of RhoA restores synaptic transmission deficits, highlighting its role as a therapeutic target.^74^ Gender differences play a significant role in 16p11.2 deletion. In a mouse model, the rate of abnormal medial fiber bundle structure was much higher in males (48%) compared to females (19%). This difference is linked to sex‐specific gene expression in the MAPK pathway, such as *MAPK3*. In male mice, *MAPK3* expression was reduced by 29%, while in female mice, it was only reduced by 12%. These findings highlight the importance of considering gender in clinical interventions. For instance, male patients with 16p11.2 deletion may require higher doses of MAPK pathway activators than female patients.^76^

The 22q11.2 deletion impacts brain connectivity and synaptic pruning by interfering with DGCR8‐mediated miRNA processing.^62^ One key gene, *DGCR8* plays a crucial role in microRNA (miRNA) biogenesis. Loss of *DGCR8* impairs miRNA processing, which disrupts synaptic plasticity and cognitive functions in mouse models.^62^, ^77^ *PRODH*, another key gene within this locus, encodes a mitochondrial enzyme involved in proline metabolism, which modulates glutamatergic neurotransmission.^78^ Alterations in *PRODH* expression are associated with disrupted synaptic transmission and plasticity, particularly in in adult mice.^78^ *PRODH*, along with *Tbx1* and Tmem10 (*T10*), is expressed during the refinement and stabilization of synaptic connections, suggesting that their loss contributes to defective synaptic development in 22q11.2 deletion syndrome.^78^ Additionally, *PRKAB2* which encodes the β subunit of the AMP‐activated protein kinase (*AMPK*) complex, plays a critical role in maintaining dendritic structure and neuronal resilience. In Drosophila, neuronal knockdown of *PRKAB2* leads to reduced dendritic arborization and progressive degeneration of dendritic structures, accompanies by a shortened lifespan and increased vulnerability to metabolic stress.^79^

The 1q21.1 deletion and duplication affect dendritic complexity and synaptic density through altered calcium channel activity.^41^ These alterations disrupt intracellular calcium homeostasis, thereby affecting activity‐dependent synaptic refinement and connectivity. Within the 3q29 CNV region, *PAK2* plays a pivotal role in synaptic development and circuit refinement by modulating cytoskeletal dynamics^68^ In mouse models, haploinsufficiency of *Pak2* results in reduced spine density and impaired synaptic plasticity, as demonstrated by defective long‐term potentiation (LTP)^68^ This is primarily attributed to disruptions in the LIMK1‐cofilin signaling pathway, which is critical for actin polymerization and synaptic structure maintenance.^68^ Additionally, Drosophila models of 3q29 deletion demonstrate that *PAK2* and *DLG2* exhibit synergistic haploinsufficiency, with co‐deletion resulting in pronounced synaptic deficits not observed when either of genes is disrupted individually.^68^, ^69^ The 7q11.23 CNV, associated with both Williams‐Beuren syndrome, disrupts neuronal excitability and connectivity *via* REST‐mediated transcriptional dysregulation, which affects the expression of key genes involved in synaptic transmission and neuronal circuit development.^80^

Several additional CNVs further illustrate the importance of synaptic pruning and connectivity. The *NRXN1* deletion disrupts trans‐synaptic signaling, disrupting the refinement of neural circuits and leading to altered synaptic organization.^70^ *CNTN4* CNVs affect axon guidance and neural circuit formation by altering cell adhesion molecule function.^70^ *SYNGAP1* deletions impact excitatory synapse maturation and pruning through dysregulation of Ras/MAPK signaling, a pathway critical for synaptic scaling and homeostasis.^71^ In summary, these genetic alterations highlight the necessity for tightly regulated synaptic pruning and connectivity in establishing functional neural networks.

### Neurotransmitter Signaling and Plasticity

CNVs perturb neurotransmitter signaling and synaptic plasticity, both of which are pivotal for adaptive neural communication and cognitive processes. Disruptions in these pathways can lead to imbalances in excitatory and inhibitory transmission, ultimately compromising synaptic adaptability and overall brain function.

The 16p11.2 deletion and duplication has been shown to affect the excitatory/inhibitory balance and synaptic plasticity through dysregulation of *MAPK3* signaling and ion channel activity.^58^ Within this locus, Doc2α, a presynaptic calcium sensor protein, plays a crucial role in maintaining synaptic integrity by regulating neurotransmitter release.^81^ Doc2α interacts with Secretagogin (SCGN), a calcium‐binding protein essential for synaptic plasticity and vesicle dynamics by facilitating spontaneous glutamate release.^81^, ^82^ Loss of Doc2α in knockout models results in reduced dendritic spine density, impaired synaptic vesicle release and abnormal neuronal morphology, ultimately leading disrupted excitatory transmission and compromised circuit formation.^81^ The 22q11.2 deletion affects dopaminergic and glutamatergic signaling, primarily through alterations in *COMT* and *PRODH* function.^62^ *COMT* encodes catechol‐O‐methyltransferase, an enzyme that degrades synaptic dopamine, and variations in its activity modulate dopaminergic signaling and cognitive performance.^83^ COMT indirectly affects synaptic plasticity by regulating dopamine metabolism: in 22q11.2 deletion mice, a 35% increase in COMT activity leads to accelerated dopamine degradation, thereby reducing postsynaptic D2 receptor activation.^33^ This effect synergizes with the abnormal glutamate release caused by *PRODH* deficiency,^84^ collectively disrupting the E/I balance — compared with single‐gene defects, double‐gene deletion increases the abnormal rate of the E/I ratio by 2.1 times.

A range of other CNVs impact neurotransmitter systems and synaptic function through diverse molecular mechanisms. The *NRXN1* deletion compromises presynaptic organization and neurotransmitter release by disrupting trans‐synaptic adhesion complexes.^70^ *GRIN2A/GRIN2B* CNVs affect NMDA receptor signaling and synaptic plasticity by altering glutamatergic transmission.^71^ *CNTNAP2* deletion impairs GABAergic signaling and neuronal excitability by disrupting K^+^ channel clustering.^70^ *SLC6A1* deletion impacts GABA reuptake and inhibitory signaling through altered GABAergic transmission. Further, the *CACNA1C* duplication affects voltage‐gated calcium channel function and calcium signaling.^72^ *SHANK3* deletion disrupts glutamatergic signaling and synaptic plasticity by interfering with postsynaptic density (PSD) organization, resulting in deficits in synaptic maturation, dendritic spine stability, and functional connectivity.^34^ In addition, *CHD8* deletion affects chromatin remodeling and synaptic gene expression by altering transcriptional regulation.^85^ Finally, the *MBD5* deletion impacts synaptic plasticity and dendritic morphology *via* altered methyl‐CpG‐binding protein function, which plays a role in transcriptional repression and chromatic state regulation.^71^ In summary, these CNVs collectively emphasize that the integrity of neurotransmitter signaling, and synaptic plasticity is indispensable for maintaining effective neural communication and cognitive resilience.

## Molecular Mechanisms of CNV‐Related Synaptic Disorders

**Fig. 3:**
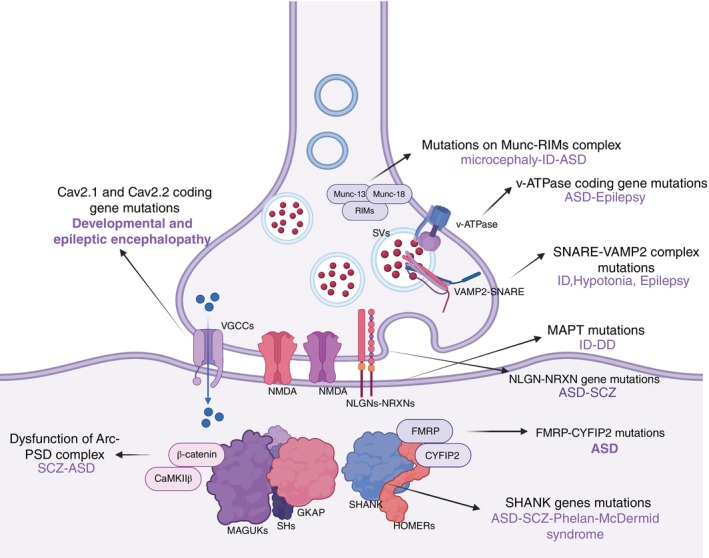
A schematic diagram illustrates the pre and post synaptic mechanisms and their associations between various gene dysfunctions and discrete neurodevelopmental disorders. On presynaptic region, mutations in the genes encoding Cav2.1 and Cav2.2 lead to developmental and epileptic encephalopathy; mutations in the Munc‐RIMs complex are associated with microcephaly, intellectual disability (ID), and autism spectrum disorder (ASD); mutations in the gene encoding v‐ATPase are linked to ASD and epilepsy; and mutations in the SNARE‐VAMP2 complex are related to ID, hypotonia, and epilepsy. On post synaptic region, mutations in the NLGN‐NRXN genes are associated with ASD and schizophrenia (SCZ); MAPT mutations are linked to ID and developmental delay (DD); FMRP‐CYFIP2 mutations are associated with ASD; SHANK gene mutations are involved in ASD, SCZ, and Phelan ‐ McDermid syndrome; and dysfunction of the Arc ‐ PSD complex is also associated with SCZ and ASD.

Neurodevelopmental disorders (NDDs), such as schizophrenia (SCZ), autism spectrum disorder (ASD), and intellectual disability (ID) is associate with synapse‐related molecular mechanisms.^86^ First, regarding the molecular bases of synapse assembly, defects in genes related to presynaptic/postsynaptic function and synaptic adhesion—including members of the NLGN and SHANK families ‐ are closely linked to NDDs.^19^, ^85^, ^87^, ^88^, ^89^, ^90^, ^91^, ^92^, ^93^, ^94^ Synapse formation is a multi‐step process, and these NDD‐risk genes along with their regulatory mechanisms may act as potential biomarkers for NDDs. Second, in presynaptic vesicle trafficking pathways, mutations in SNARE complex members (e.g., *SNAP25, VAMP2*) cause various NDDs, while proteins like SYT1 and Munc18 (which assist in neurotransmitter release) also lead to disorders when mutated.^7^, ^95^, ^96^, ^97^, ^98^, ^99^ Additionally, mutations in genes involved in synaptic vesicle retrieval (e.g., dynamin‐1) disrupt synaptic activity, and other genes such as FMRP contribute to NDDs by impacting vesicle function.^7^, ^22^, ^100^, ^101^ Finally, for postsynaptic plasticity and signaling, NDD‐risk genes are categorized into two groups—those regulating transcription/epigenetics and those mediating neurotransmission—alterations in postsynaptic density (PSD) complex genes (e.g., PSD‐95) are associated with SCZ, ASD, and more (with overlapping risk genes between SCZ and ASD), and chronic stress further impairs synaptic structure and function.^102^, ^103^, ^104^ (Fig. 3).

Synaptic dysfunction in neurodevelopmental disorders associated with rare CNVs arises from multifaceted impairments spanning synaptic transmission, cellular energetics, and molecular signaling cascades.

### Disrupted Excitatory/Inhibitory (E/I) Balance

The homeostatic balance between excitatory (glutamatergic) and inhibitory (GABAergic) synaptic transmission is fundamental to normal neuronal circuitry. In CNV‐associated disorders, this balance is perturbed *via* alterations in AMPA/GABA receptor‐mediated signaling and glutamatergic plasticity pathways (e.g., NMDA/AMPA receptor‐dependent long‐term potentiation, LTP).^102^ For example, 22q11.2 deletion models exhibit reduced GABAergic synaptic density, shifting the E/I balance toward excitation^105^ while 16p11.2 duplication models show exaggerated glutamatergic transmission, leading to network hyperexcitability.^106^ Such imbalances directly disrupt information processing in cortical and limbic circuits, contributing to cognitive and behavioral deficits. These E/I imbalances are often driven by calcium signaling defects: in CNV models (e.g., 1q21.1 deletion), NMDA receptor‐mediated calcium influx is reduced by 40%, which not only impairs glutamatergic synaptic plasticity but also disrupts the maturation of GABAergic interneurons—creating a feedforward loop of E/I dysregulation.^31^

### Mitochondrial and RNA‐Dependent Mechanisms Beyond Synaptic Proteins

Synaptic function is also modulated by non‐synaptic protein mechanisms. Neurons depend on mitochondria to meet the high ATP demand for synaptic transmission (e.g., neurotransmitter release, ion pump activity). In ASD and CNV models (e.g., 22q11.2 deletion), mitochondrial abnormalities—such as impaired oxidative phosphorylation and calcium buffering—compromise synaptic integrity.^107^, ^108^ For instance, 22q11.2 deletion cortical organoids display mitochondrial fragmentation in presynaptic terminals, reducing vesicle recycling efficiency.^55^ Processes like alternative splicing, transcription, and editing dynamically regulate synaptic protein expression. In 15q11‐13 duplication models, dysregulated RNA editing of glutamate receptor subunits alters synaptic plasticity,^106^ while 16p11.2 deletion disrupts the translation of PSD‐95 *via* FMRP‐dependent mechanisms.^22^

### CNV‐Specific Synaptic Pathway Impairments

Synaptic deficits, both shared and unique, have been observed across neurodevelopmental CNVs such as 22q11.2, 16p11.2, 1q21.1, and 3q29. These CNVs cause synaptic deficits through three main mechanisms. First, gene dosage imbalance (e.g., haploinsufficiency of SHANK3 in 22q11.2 deletion); second, disruption of topologically associated domain (TAD) boundaries, leading to ectopic expression of synaptic genes like NRXN1; and third, maternal epigenetic transmission (e.g., DNA methylation marks) that disrupts synaptic development even without direct CNV inheritance.^38^ These upstream genetic‐epigenetic disruptions lay the foundation for CNV‐specific synaptic deficits.

22q11.2 deletion models, including thalamocortical organoids, exhibit abnormal axonal outgrowth. In mice, this deletion also causes dendritic spine defects, with a 22% reduction in length and an 18% decrease in density, attributed to mTOR pathway inhibition.^109^ Crucially, restoring mTOR activity with rapamycin analogs not only rescues spine morphology but also improves social memory in these mice, confirming mTOR‐mediated spine defects as a causal factor in behavioral phenotypes.^109^ Schizophrenia‐derived cortical neurons further show reduced dendritic spine density.^110^, ^111^ 22q11.2 deletion models (mouse and organoid) display reduced calcium transient amplitude,^53^, ^55^ 16p11.2 duplication neurons exhibit dysregulated calcium buffering; and 1q21.1 deletion excitatory neurons show aberrant network calcium activity.^41^, ^112^ Furthermore, mTOR‐dependent pathology in 16p11.2 duplication drives excessive excitatory synaptic density and functional hyperconnectivity.^113^ Human iPSC‐derived neurons with 16p11.2 CNV show coordinated pre‐ and postsynaptic defects: a 35% reduction in presynaptic glutamate release frequency and a 28% downregulation of postsynaptic PSD‐95 expression, synergistically impairing synaptic transmission.^114^ Similarly, 22q11.2 deletion and 15q11‐13 duplication models exhibit E/I imbalance and disrupted synaptic transmission.^105^, ^106^, ^115^, ^116^ Recent studies also demonstrate that microglia with 1q21.1 deletion and duplication have a compromised ability to activate and display an impaired inflammatory profile.^117^ This suggests these microglia have a limited capacity to perform physiological functions like synaptic pruning, which can in turn influence neuronal function. Collectively, these findings (summarized in Table 2) underscore the pleiotropic effects of CNVs on synaptic machinery, manifesting as both shared features (e.g., calcium and E/I dysregulation) and unique, cell‐type‐specific defects across different disorders.

**Table 2:** Diverse CNVs, AD and SCZ and their effects on neuronal and synaptic networks on cellular and mouse mode

CNVs	Model	Impact on Synaptic development/function	Reference
22q11.2 Deletion	Organoid model	Four times higher spontaneous firing compared to control Decrease on calcium amplitude Enrichment on genes relating to mitochondrial function, neuronal excitability, calcium transport and signaling, voltage gated calcium channel activity	^53^
22q11.2 Deletion	Organoid model	Transcriptomic changes on neurodevelopmental disorder risk genes Abnormal axonal outgrowth	^111^
22q11.2 Deletion	Mouse model	Significantly altered calcium amplitude and calcium signaling dysregulation Alterations on inhibitory synaptic events Changes on mRNA expression on ion channel receptors and transporters Alterations on genes relating to neuronal activity Abnormal calcium homeostasis	^53^
22q11.2 deletion	IPSC model	Reduction on neurite outgrowth and cellular migration Dysregulated balance of neurogenic to gliogenic competence	^118^
22q11.2 deletion	IPSC model	Reduction on spiking rate Reduction on neuronal network activity Transcriptional and translational level of changes on ASD/SCZ risk genes including NRXN1	^119^
22q11.2 deletion	Mouse model	Reduction on GABA receptor levels and dysregulation on inhibitory and excitatory neuronal networks	^105^
22q11.2 deletion	Mouse model	Impairments on functional connectivity and alterations on dendritic spine density	^120^
22q11.2 deletion	Mouse and human model	Impairments on mitochondrial transporter system to provide synaptic function	^121^
16p11.2 duplication	IPSC model	Reduction on mean firing rate, synchronicity, burst frequency on neuronal network activity Enrichment on SCZ/ASD risk genes Enrichment of genes relating to glutamargic synapses Impairment on calcium homeostasis	^112^
16p11.2 duplication	Mouse model	Increased mature oligodendrocytes, hypermyelination, more myelinated axons; increased axon diameter with irregular morphology; sustained astrocyte activation	^122^
16p11.2 deletion and 16p11.2 duplication	IPSC and organoid model	Neurons showed reduced firing rate, synchrony and oscillation Whereas 16p del organoids displayed more inhibitory neurons, 16p dup organoids displayed more excitatory neurons	^51^
16p11.2 deletion	Mouse model	Enhancement on the ratio of AMPA to NMDA receptor mediated excitatory post synaptic currents	^115^
16p11.2 deletion	Mouse model	Male specific structural changes on medial fiber tracts and their overlapping gene expression patterns relating to neurite outgrowth and MAPK pathway	^76^
16p11.2 duplication	Mouse model	Hyperexcitability with significant defects on GABAergic synaptic transmission	^116^
16p11.2 deletion and 16p11.2 duplication	IPSC model	Less excitatory synapses with enhanced synaptic strength Alterations on neuronal activity	^58^
16p11.2 deletion and 16p11.2 duplication	Organoid model	Impairment on neuronal migration	^27^
16p11.2 deletion	Mouse model	Alterations on synaptic transmission and neuronal maturation	^43^
16p11.2 deletion	Mouse model	Alterations on functional connectivity and GABAergic dysfunction	^123^
16p11.2 deletion	Mouse model	Reduced mature oligodendrocytes, mild hypomyelination, fewer myelinated axons; increased axon diameter; astrocyte activation (P21) followed by reduction (P90)	^122^
1q21.1 deletion	Mouse model	Enhancement on dopamine cell firing and neuronal activity Increased sensitivity to amphetamine	^124^
1q21.1 deletion and 1q21.1duplication	IPSC model	Aberrations on neuronal network and calcium activity Impairment on synaptogenesis	^41^
3q29 deletion	Mouse model	Enhancement on excitatory neuronal activity in cerebral cortex	^67^
2p16.3 deletion	IPSC model	Enhancement on frequency, duration and amplitude of calcium transients of cortical pyramidal neurons	^125^
2p16.3 deletion	IPSC model	Increased neuronal excitability, action potential amplitude with faster depolarisation	^126^
2p16.3 deletion	IPSC model	Reduction on the frequency of excitatory postsynaptic currents and AMPA mediated postsynaptic current amplitude with decreased neurotransmitter release	^127^
2p16.3 deletion	Organoid model	Reduction on the frequency of spontaneous calcium transients and synchronous firing rate	^128^
15q11‐13 duplication; Angelman	IPSC model	Neuronal hyperexcitability, increased synaptic event frequency and amplitude, increased frequency of action potential firing, reduction on inhibitory synaptic transmission	^106^
Schizophrenia	Mouse model	Selective ablation of NMDA receptors in cortical and hippocampal interneurons which resulting E/I imbalances of cortical synaptic inputs	^129^
Schizophrenia	IPSC model	Significant decline on dendritic spine density in schizophrenia cortical neurons	^110^
Schizophrenia	IPSC model	Alterations on synaptic function involving enhancement on glutamargic synaptic transmission, higher synaptic density	^130^
Autism spectrum disorder	Mouse model	Synaptic defects and impairments on neuronal circuits	^131^
Autism spectrum disorder	IPSC model	Early maturation and hyperexcitability	^132^
Autism spectrum disorder	Mouse model	Enhancement on excitatory synaptic density with mTOR related pathology and functional hyperconnectivity	^113^
Autism spectrum disorder	Mouse model	Alterations on synaptic protein expressions and abnormal spine morphology	^133^

### Therapeutic interventions in CNV models

A wide range of drug discovery efforts have focused on targeting postsynaptic dysfunction, with many of these interventions also unexpectedly improving presynaptic activity. One example is arbaclofen, a potent GABA_B receptor agonist, which activates potassium channels to restrict calcium influx through NMDA receptors, thereby alleviating synaptic impairment.^134^ Similarly, the BK channel opener BMS‐204352 and the Kv7 channel agonist retigabine have demonstrated promising effects by enhancing neurotransmitter release and supporting synaptic function.^135^, ^136^ In experimental research, ketamine is commonly used to induce psychotic‐like experiences, making it a valuable tool for generating animal models of psychosis and investigating the underlying mechanisms of synaptic dysfunction.

As outlined earlier, several copy number variation (CNV) syndromes are linked to neurodevelopmental and neuropsychiatric disorders. Most therapeutic approaches focus on drug treatments targeting neuropsychiatric symptoms caused by CNVs, while fewer strategies address downstream effects, such as disruptions in signaling pathways, cellular functions, synaptic activity, and neuronal circuits. Identifying specific pathway targets is challenging because large, common CNVs involve multiple genes with diverse functions and interconnected mechanisms. Nevertheless, certain synaptic signaling pathways, as well as calcium and potassium channel modulators, have shown potential for reversing disease phenotypes. For example, the BK channel opener BMS‐204352 and the Kv7 channel agonist retigabine have demonstrated promise in enhancing neurotransmitter release and supporting synaptic function.^135^, ^136^

The 22q11.2 and 16p11.2 deletion or duplication models are the most commonly used CNVs in therapeutic studies involving mouse and cellular research. Overactivation of the RhoA/Rho‐kinase pathway is linked to disruptions in key cellular functions, such as apoptosis and proliferation, and contributes to oxidative stress.^57^ Therefore, RhoA inhibitors Rhosin and Fasudil have been used to rescue some phenotypes of 16p11.2 deletion and have a substantial effect on reducing hyperactivity, reverse abnormal cytostructural and neuronal migration and ameliorated some behavioral phenotypes on mouse and cellular studies.^27^, ^137^, ^138^ Another study combined RhoA inhibitor with Ras–ERK pathway inhibitors as ERK pathway is essential to provide neuronal development, function and plasticity and they revealed significant reduction on hyperactivity and reversed behavioral phenotypes with memory defects.^137^, ^139^ Impairments on cellular and synaptic signaling pathways represent one of the factors underlying neurodevelopmental disorders. Blizinski *et al*. demonstrated the ameliorative effect of MEK/ ERK pathway inhibitor U0126 on 16p11.2 duplication mouse cortical neurons and they identified the inhibition on excessive dendritic branching.^140^ A wide range of drug discovery efforts have focused on targeting postsynaptic dysfunction, with many of these interventions also unexpectedly improving presynaptic activity. One example is arbaclofen, a potent GABA_B receptor agonist, which activates potassium channels to restrict calcium influx through NMDA receptors, thereby alleviating synaptic impairment.^134^ Defects on NMDA/AMPA receptors and so neurotransmission is another contributing factor to lead neurodevelopmental disorder phenotypes and one of the attractive studies, GABA receptor agonist arbaclofen have been used on 16p11.2 deletion mouse model and reversed social and cognitive phenotype.^141^ In the same context, negative allosteric modulator of metabotropic glutamate receptor 5 CTEP have been induced on 16p11.2 deletion mouse model and ameliorated some behavioral phenotype.^142^ So far, only two drug studies have published for 22q11.2 deletion syndrome and Donegan *et al*. used potassium channel TREK‐1 antagonist Spadin as reducing neuronal firing and ameliorating memory and another study induced dopamine D2/D3 receptor antagonist Raclopride on human cortical neurons to reverse abnormal inactivation of voltage gated calcium channels.^55^, ^143^ Targeting the core defects of 16p11.2 deletion—excessive activation of RhoA and inhibition of the ERK pathway, Pucilowska *et al*. (2018)^137^ used a combination of the RhoA inhibitor Fasudil and an ERK pathway activator in mice, rescuing dendritic spine density and memory deficits, with effects verified in iPSC‐derived neurons.^138^ This suggests that dual‐target intervention can synergistically reverse synaptic structural and functional defects. Abnormalities in voltage‐gated calcium channels caused by 22q11.2 deletion can be corrected by the D2/D3 receptor antagonist Raclopride: Khan *et al*. (2020)^55^ treated human cortical organoids with Raclopride, restoring depolarization‐activated calcium influx and synaptic vesicle release. This effect is complementary to the result that the TREK‐1 antagonist Spadin improved firing in the hippocampal CA2 region in mouse models.^143^

Research on the other CNVs covered in this review is limited. Only one drug study for the 1q21.1 deletion targeted voltage‐gated calcium channels. Using the channel blocker Verapamil, researchers successfully modulate calcium activity.^41^ Notably, copy number variations (CNVs) in synaptic pathways can predict therapeutic responses. For example, in schizophrenia patients, deletions of CNVs in KEGG synaptic signaling pathways are linked to a 30% greater reduction in PANSS scores after antipsychotic treatment. Meanwhile, CNVs in xenobiotic metabolism pathways can also affect a drug's efficacy.^144^ CNV profiling shows promise for guiding personalized treatment of CNV‐associated neurodevelopmental disorders. For example, two separate studies on 3q29 mice demonstrated that antipsychotic Risperidone and Oxytocin rescued distinct behavioral phenotypes. Furthermore, ketamine is often used to induce psychotic‐like experiences, making it a useful tool for creating animal models of psychosis and studying the mechanisms of synaptic dysfunction.^67^, ^145^ Table 3 displays all CNV drug treatment studies, including their strategies and models.

**Table 3:** Pharmacological rescue strategies in various copy number variant models

CNVs	Model System	Drugs	Pathway (Detailed Mechanism)	Rescued Phenotype	Reference
16p11.2 deletion	Mouse	Fasudil	Inhibitor of Rho‐associated protein kinase (ROCK), Modulates Ras–ERK signaling pathway	Reverse abnormal cytoarchitecture, behavioral phenotype, hyperactivity, and impaired memory	^137^
	Mouse	Fasudil	Inhibitor of Rho‐associated protein kinase (ROCK), Modulates Ras–ERK signaling pathway	Object recognition memory and learning	^146^
	Human induced dopaminergic neurons	Rhosin	Specific RhoA inhibitor	Hyperactivity and soma size	^138^
	Mouse	CTEP	Negative allosteric modulator of mGluR5	Fear conditioning	^142^
	Mouse	R‐baclofen	GABAB receptor agonist	Social and cognitive phenotype	^141^
	Mouse	MS‐275 or Romidepsin	Class I histone deacetylase (HDAC) inhibitor	Hyperactivity of fast spiking interneurons, NMDA and GABA mediated synaptic currents	^147^
16p11.2 duplication	Mouse cortical neurons	U0126	MEK–ERK pathway inhibitor	Cortical neuronal arborization as inhibiting excessive dendritic branching	^140^
	Human cortical organoids	Rhosin	RhoA inhibitor	Neuronal migration	^27^
22q11.2 deletion	Mouse	Spadin	TREK‐1 antagonist	Hippocampal CA2 firing and social memory	^143^
	Human cortical organoids	Raclopride	D2/D3 receptor antagonist	Depolarization activated calcium influx	^55^
1q21.1 deletion	Human induced cortical neurons	Verapamil	Voltage‐gated calcium channel (VGCC) blocker	Calcium activity	^41^
3q29 deletion	Mouse	Oxytocin	Oxytocin receptor agonist	Social behavior	^145^
	Mouse	Risperidone	Atypical antipsychotic; D2/5‐HT2A antagonist	Startle amplitude, pre‐pulse inhibition	^67^
22q13 deletion	Human induced cortical neurons	IGF1	Activates PI3K/AKT/mTOR and MAPK/ERK pathways	Number of excitatory synapses and excitatory synaptic transmission	^46^
7q11.23 deletion	Mouse	JZL184	Endocannabinoid pathway (FAAH inhibitor)	Short‐term memory	^148^
	Mouse	Epigallocatechin‐3‐gallate (green tea)	Polyphenolic antioxidant; modulates synaptic signaling	Social and cognitive phenotype	^149^
	Mouse	Verapamil	Voltage‐gated calcium channel (VGCC) blocker	Social behavior	^109^
15q11‐13 duplication	Mouse	Fluoxetine	Serotonin reuptake inhibitor	Social phenotype and GABAergic synaptic function	^150^
	Mouse	Oxytocin or 8OH‐DPAT	5‐HT1A receptor agonist	Social behavior	^151^
15q13.3 deletion	Mouse	Lu AF58801	Positive allosteric modulator of nAChA7R	Functional connectivity patterns	^152^
Rett syndrome	Human induced cortical neurons and organoids	Nefiracetam and/or PHA 543613	Ampakine (AMPA receptor modulator)	Reversed network pathology/synaptic gene expression	^153^
Angelman syndrome	Mouse	5‐HT7R	Serotonin receptor	Rescue of dendritic spine density	^154^

## Conclusion

CNVs contribute to synaptic dysfunction based on a complex interplay of divergent and convergent mechanisms. Each CNV discussed in this review perturbs distinct upstream molecular pathways, from miRNA biogenesis and cytoskeletal regulation to MAPK/ERK signaling and synaptic protein trafficking. These divergent mechanisms reflect the breadth of genetic vulnerabilities impacting synaptic development.

However, a convergence emerges at the level of synaptic pathology. Almost universally, CNVs implicated in neurodevelopmental disorders disrupt key cellular processes fundamental to synaptic function, including cytoskeletal dynamics, dendritic spine morphology, synaptic plasticity, and the balance of excitatory and inhibitory transmission. These disruptions compromise neuronal activity and circuit integrity, contributing to shared neurodevelopmental phenotypes such as cognitive deficits, autism spectrum disorder and schizophrenia.

Recognizing this convergence is crucial for future therapeutic strategies. By targeting these common downstream processes, rather than the highly diverse upstream genetic variations, there is potential to develop interventions with broader efficacy across CNV‐associated disorders. Future research should aim to further delineate these shared pathogenic nodes, providing a foundation for unified, mechanism‐based treatments.

## Author contributions

Tianqi Wang: Original draft writing, review, editing, and illustration; Kubra Trabzonlu: Original draft writing, review, editing, and illustration; Emily Fullard Jones: Review and editing; Yasir Ahmed Syed: Conceptualization, original draft writing, review, and editing.

## Disclosure statement

The authors declare no potential conflicts of interest.

## Data Availability

Data sharing not applicable to this article as no datasets were generated or analysed during the current study.
